# Supplementary material for: Discriminant Canonical Analysis as a Validation Tool for Multivariety Native Breed Egg Commercial Quality Classification
Source: Foods. 2021 Mar 17;10(3):632. doi: 10.3390/foods10030632 (PMC8002516; doi:10.3390/foods10030632)
Supplement: Supplementary file 1 [file foods-10-00632-s001.zip › Table S1.docx]

|  | White L | White M | White S | White XL | Franciscan L | Franciscan M | Franciscan S | Franciscan XL | Leghorn L | Leghorn M | Leghorn S | Leghorn XL | Black L | Black M | Black S | Black XL | Partridge L | Partridge M | Partridge S | Partridge XL |
| --- | --- | --- | --- | --- | --- | --- | --- | --- | --- | --- | --- | --- | --- | --- | --- | --- | --- | --- | --- | --- |
| White L | 80 | 0 | 0 | 0 | 6.7 | 0 | 0 | 0 | 6.7 | 0 | 0 | 0 | 3.3 | 0 | 0 | 0 | 3.3 | 0 | 0 | 0 |
| White M | 4.8 | 47.6 | 0 | 0 | 0 | 14.3 | 0 | 0 | 0 | 23.8 | 4.8 | 0 | 0 | 4.8 | 0 | 0 | 0 | 0 | 0 | 0 |
| White S | 0 | 0 | 100 | 0 | 0 | 0 | 0 | 0 | 0 | 0 | 0 | 0 | 0 | 0 | 0 | 0 | 0 | 0 | 0 | 0 |
| White XL | 0 | 0 | 0 | 50 | 0 | 0 | 0 | 0 | 50 | 0 | 0 | 0 | 0 | 0 | 0 | 0 | 0 | 0 | 0 | 0 |
| Franciscan L | 25 | 0 | 0 | 0 | 33.3 | 8.3 | 0 | 0 | 8.3 | 0 | 0 | 0 | 8.3 | 0 | 0 | 0 | 16.7 | 0 | 0 | 0 |
| Franciscan M | 0 | 0 | 2.4 | 0 | 0 | 76.2 | 0 | 0 | 0 | 11.9 | 0 | 0 | 0 | 4.8 | 0 | 0 | 2.4 | 2.4 | 0 | 0 |
| Franciscan S | 0 | 0 | 0 | 0 | 0 | 0 | 100 | 0 | 0 | 0 | 0 | 0 | 0 | 0 | 0 | 0 | 0 | 0 | 0 | 0 |
| Franciscan XL | 0 | 0 | 0 | 0 | 0 | 0 | 0 | 100 | 0 | 0 | 0 | 0 | 0 | 0 | 0 | 0 | 0 | 0 | 0 | 0 |
| Leghorn L | 2.1 | 0 | 0 | 0 | 0 | 2.1 | 0 | 0 | 87.5 | 8.3 | 0 | 0 | 0 | 0 | 0 | 0 | 0 | 0 | 0 | 0 |
| Leghorn M | 0 | 0 | 0 | 0 | 0 | 2.1 | 2.1 | 0 | 4.2 | 89.6 | 0 | 0 | 2.1 | 0 | 0 | 0 | 0 | 0 | 0 | 0 |
| Leghorn S | 0 | 0 | 0 | 0 | 0 | 12.5 | 0 | 0 | 0 | 0 | 75 | 0 | 0 | 0 | 0 | 0 | 0 | 0 | 12.5 | 0 |
| Leghorn XL | 0 | 0 | 0 | 0 | 0 | 0 | 0 | 0 | 26.7 | 0 | 0 | 73.3 | 0 | 0 | 0 | 0 | 0 | 0 | 0 | 0 |
| Black L | 12.5 | 0 | 0 | 0 | 0 | 6.3 | 0 | 0 | 12.5 | 0 | 0 | 6.3 | 50 | 0 | 0 | 0 | 12.5 | 0 | 0 | 0 |
| Black M | 0 | 0 | 0 | 0 | 0 | 12.5 | 0 | 0 | 0 | 6.3 | 0 | 0 | 0 | 75 | 6.3 | 0 | 0 | 0 | 0 | 0 |
| Black S | 0 | 0 | 0 | 0 | 0 | 0 | 0 | 0 | 0 | 0 | 0 | 0 | 0 | 25 | 75 | 0 | 0 | 0 | 0 | 0 |
| Black XL | 0 | 0 | 0 | 0 | 0 | 0 | 0 | 0 | 0 | 0 | 0 | 14.3 | 0 | 0 | 0 | 85.7 | 0 | 0 | 0 | 0 |
| Partridge L | 11.1 | 0 | 0 | 0 | 16.7 | 5.6 | 0 | 0 | 0 | 0 | 0 | 0 | 0 | 0 | 0 | 0 | 61.1 | 0 | 0 | 5.6 |
| Partridge M | 9.1 | 9.1 | 0 | 0 | 0 | 45.5 | 0 | 0 | 0 | 9.1 | 0 | 0 | 0 | 9.1 | 0 | 0 | 0 | 18.2 | 0 | 0 |
| Partridge S | 0 | 0 | 0 | 0 | 0 | 0 | 0 | 0 | 0 | 0 | 0 | 0 | 0 | 0 | 0 | 0 | 0 | 0 | 100 | 0 |
| Partridge XL | 0 | 0 | 0 | 0 | 0 | 0 | 0 | 0 | 0 | 0 | 0 | 0 | 0 | 0 | 0 | 0 | 0 | 0 | 0 | 100 |

**Table S1.** Appropriately classified eggs according to the commercial size and genotype of the laying hen.
